# MicroRNA-32 (miR-32) regulates phosphatase and tensin homologue (PTEN) expression and promotes growth, migration, and invasion in colorectal carcinoma cells

**DOI:** 10.1186/1476-4598-12-30

**Published:** 2013-04-23

**Authors:** Weiyun Wu, Jingfang Yang, Xiao Feng, Hao Wang, Shicai Ye, Pengchun Yang, Wenkai Tan, Guoli Wei, Yu Zhou

**Affiliations:** 1 Department of Gastroenterology, The affiliated Hospital of Guangdong Medical College, South Peoples Avenue No. 57, Xiashan District, Zhanjiang, Guangdong, China

**Keywords:** microRNA, Colorectal carcinoma, PTEN, Invasion

## Abstract

**Background:**

Colorectal carcinoma (CRC) is one of the leading causes of cancer-related mortality worldwide. MicroRNAs (miRNAs, miRs) play important roles in carcinogenesis. MiR-32 has been shown to be upregulated in CRC. In this study, we identified the potential effects of miR-32 on some important biological properties of CRC cells, and clarified the regulation of PTEN by miR-32.

**Methods:**

The effect of miR-32 on PTEN expression was assessed in CRC cell lines with miR-32 mimics/inhibitor to increase/decrease miR-32 expression. Furthermore, the roles of miR-32 in regulating CRC cells biological properties were analyzed with miR-32 mimics/inhibitor-transfected cells. The 3^′^-untranslated region (3^′^-UTR) of PTEN combined with miR-32 was verified by dual-luciferase reporter assay.

**Results:**

Gain-of-function and loss-of-function studies showed that overexpression of miR-32 promoted SW480 cell proliferation, migration, and invasion, reduced apoptosis, and resulted in downregulation of PTEN at a posttranscriptional level. However, miR-32 knock-down inhibited these processes in HCT-116 cells and enhanced the expression of PTEN protein. In addition, we further identified PTEN as the functional downstream target of miR-32 by directly targeting the 3^′^-UTR of PTEN.

**Conclusions:**

Our results demonstrated that miR-32 was involved in tumorigenesis of CRC at least in part by suppression of PTEN.

## Introduction

Colorectal carcinoma (CRC) is one of the most common cancers, and is a significant contributor to cancer death [1]. CRC carcinogenesis is a multi-step process in which a normal cell undergoes malignant transformation to a fully developed tumor through accumulations of genetic and epigenetic changes. Although a number of molecular events have been identified, more and more new molecules that play a role in this process remain to be discovered, which are crucial for development of improved therapeutic approaches. Thus, a deeper understanding of the molecular and genetic networks that control the initiation and progression of CRC is imperative.

MicroRNAs (miRNAs, miRs) are small non-coding RNAs that regulate gene expression by the inhibition of the translation and/or decreasing of the stability of target mRNAs [2]. MicroRNAs participate in gene regulation, apoptosis, hematopoietic development, the maintenance of cell differentiation, and tumor genesis [3]. Recent data suggest that dysregulation of miRNAs is an important step in the pathogenesis, from initiation to metastasis, of many cancers including CRC [4-6]. The dysregulation of miRNA expression is associated with oncogenic transformation. MicroRNAs that act as tumor suppressors (e.g., miR-145, miR-124 and miR-142-3p) [7-9] or oncogenes (e.g., miR-21, miR-218, and miR-24) [10-12] have been identified in many types of tumors. Strillacci et al. [13] reported an inverse correlation between COX-2 and miR-101 expression in colon cancer cell lines, and demonstrated the direct inhibition of COX-2 mRNA translation mediated by miR-101. Shen et al. [14] found that miR-139 inhibits invasion and metastasis of CRC by targeting the type I insulin-like growth factor receptor. Recently, Sarver et al. [15] using microarray analysis had shown that miR-32 was upregulated in CRC. In their study, the authors quantified the expression levels of 735 miRNAs in 80 human CRC samples and 28 normal colon tissues, and identified 39 miRNAs, including miR-32, whose expression levels were significantly altered in CRC samples. However, the function of miR-32 in CRC remains unknown.

The phosphatase and tensin homologue (PTEN) protein is a well-known anti-oncogene. PTEN is one of the most frequently mutated tumor suppressors in a variety of human cancers [16-18]. Its loss of expression is associated with tumor progression and poor clinical outcome in CRC [19]. Nuclear PTEN expression gradually decreases during the normal-adenoma-adenocarcinoma sequence, which suggests an important role for PTEN in carcinogenesis [20]. PTEN is a negative regulator of the PI3K/Akt pathway [21], and the PTEN loss-PI3K/pAkt pathway may play an important role in sporadic colon carcinogenesis. Reduction of PTEN expression may predict relapse in CRC patients [22]. Bioinformatics has shown that the 3^′^-UTR of PTEN contains a putative binding site for miR-32. However, the regulation of miR-32 in CRC or it association with PTEN have not been reported.

In this study, we focused on the expression and function of miR-32 in CRC cells. In gain-of-function and loss-of-function studies, we found that miR-32 promoted CRC cells growth, migration, invasion, and reduced apoptosis. Overexpression of miR-32 resulted in downregulation of PTEN at a posttranscriptional level. By using a luciferase-reporter gene, we identified PTEN as the functional downstream target of miR-32.

## Results

### Expression of miR-32 in CRC cell lines

**Figure 1:**
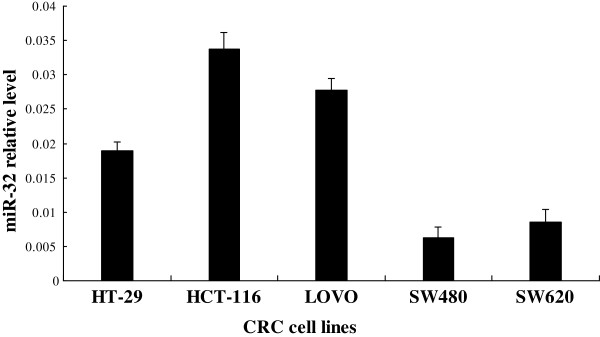
**qRT-PCR of miR-32 expression in CRC cell lines with different degrees of differentiation and metastasis ability.** Data were the means of three measurements and the bars represented SD of the mean.

We first analyzed the expression level of miR-32 in a panel of CRC cell lines with different degrees of differentiation and metastatic ability including LOVO (undifferentiated), HT-29 (high differentiation), HCT-116 (low differentiation), SW480 (low metastatic ability), SW620 (high metastatic ability). We observed that miR-32 expression was relatively higher in HCT-116 cells than in HT-29 cells, and also was lower in SW480 cells than in SW620 cells (Figure 1), suggesting that miR-32 expression may be associated with the degree of CRC cell differentiation and metastatic ability. Based on this expression pattern, we therefore chose SW480 and HCT-116 cells for the following gain-of-function and loss-of-function studies, respectively.

### MiR-32 binds to the 3^**′**^-UTR of PTEN

**Figure 2:**
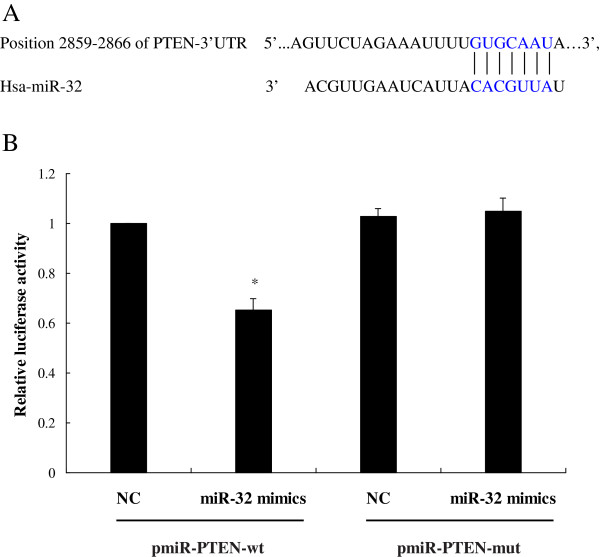
**MiR-32 and 3**^**′**^**-UTR of PTEN.** (**A**) Position of the miR-32 target site in 3^′^-UTR of PTEN mRNA predicted by TargetScan. (**B**) Effect of miR-32 on PTEN expression by luciferase reporter assay. SW480 cells were cotransfected with miR-32 mimics(or miR-32 mimics-NC) with pmiR-PTEN-wt (or pmiR-PTEN-mut) vector. Luciferase activity was normalized by the ratio of firefly and Renilla luciferase signals. The results were expressed as fold change relative to the negative control.*P < 0.05 when compared to other three groups. Data were the means of three measurements and the bars represented SD of the mean.

Analysis by using publicly available programs, TargetScan (http://www.targetscan.org) and miRanda (http://www.microrna.org), indicates that PTEN is theoretically the target gene of miR-32 (Figure 2A). We then performed a luciferase reporter assay to verify that miR-32 directly targets PTEN. We found that co-transfection of miR-32 mimics and pmiR-PTEN-wt significantly decreased the luciferase activity in SW480 cells as compared with the control. However, miR-32 mimics had no effect on the luciferase activity when co-transfected with pmiR-PTEN-mut (Figure 2B). These data showed that PTEN is one of direct targets of miR-32.

### Alteration of miR-32 expression changed PTEN protein expression but not mRNA level

**Figure 3:**
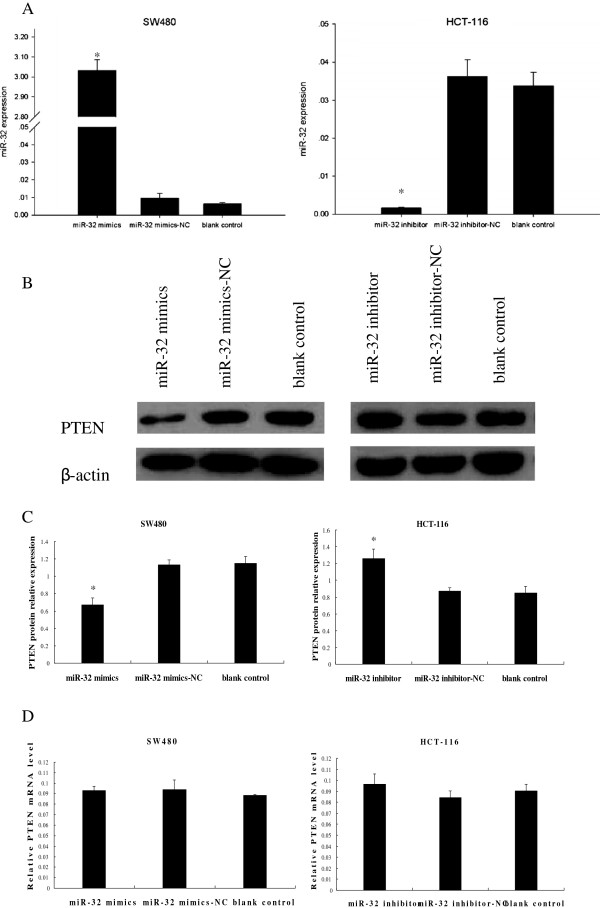
**Alteration of miR-32 expression changes PTEN protein expression.** (**A**) MiR-32 expression was detected by qRT-PCR. SW480 cells transfected with miR-32 mimics showed an increase in miR-32 expression, while HCT-116 cells transfected with miR-32 inhibitor resulted in significantly decreased miR-32 expression. *P < 0.05 when compared with corresponding negative control. (**B**) Representative pictures of PTEN protein expression detected by western blot. (**C**) Quantitative analysis showed that PTEN protein expression in SW480 cells transfected with miR-32 mimics decreased when compared with miR-32 mimics-NC or blank control. PTEN protein increased in miR-32 inhibitor transfected HCT-116 cells when compared with miR-32 inhibitor-NC or blank control. *P < 0.05 (**D**) Transfected cells had no alternation in PTEN mRNA level. Data were the means of three measurements and the bars represented SD of the mean.

PTEN had been reported to regulate CRC carcinogenesis [19]. To further confirm that PTEN was the downstream target of miR-32, up-regulation and down-regulation of miR-32 expression were conducted with subsequent detection of PTEN mRNA and protein change. Compared to miR-32 mimics-NC or blank control (without transfection), transfection with 100 nM of miR-32 mimics in SW480 cells led to an approximately 300-fold increase in miR-32 expression as detected by qRT-PCR (Figure 3A). The increase in endogenous miR-32 levels significantly decreased PTEN protein expression as determined by western blot (*P* < 0.05) (Figure 3B, C), while mRNA remained unchanged (*P* > 0.05) (Figure 3D). In contrast, to conduct loss-of-function experiments 150 nM of miR-32 inhibitor was transfected into HCT-116 cells and compared to miR-32 inhibitor-NC or blank control. The results showed a decrease of miR-32 expression (Figure 3A) and an increase PTEN protein expression (*P* < 0.05) (Figure 3B, C) with no mRNA alternation (*P* > 0.05) (Figure 3D).

### MiR-32 promoted CRC cell proliferation

**Figure 4:**
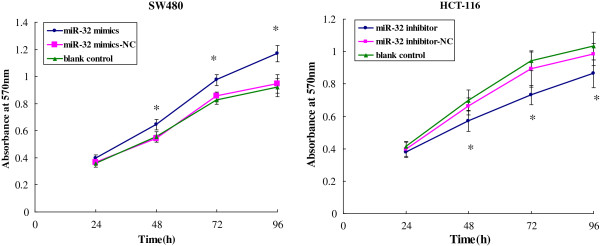
**The viable cell number was evaluated as the value of the absorbance at 570 nm.** Transfection with miR-32 mimics promoted SW480 cells proliferation and transfection with miR-32 inhibitor inhibited HCT-116 cells proliferation compared to the corresponding control at different time points. *P < 0.05, as compared miR-32 mimics or inhibitor with other two corresponding groups at the indicated time points. Data were the means of three measurements of OD and the bars represented SD of the mean.

MiR-32 has been reported to be upregulated in CRC by miRNA microarray analysis [15], implicating its potential role in CRC cells biological properties. To further characterize the functional importance in CRC tumorigenesis, we examined the effect of miR-32 on the proliferation of CRC cells using MTT assay. We observed that over-expression of miR-32 significantly promoted the proliferation of SW480 cells, whereas miR-32 inhibition restrained the proliferation of HCT-116 cells at 48, 72, 96 h after transfection, respectively (*P* < 0.05) (Figure 4).

### MiR-32 reduced apoptosis in CRC cells

**Figure 5:**
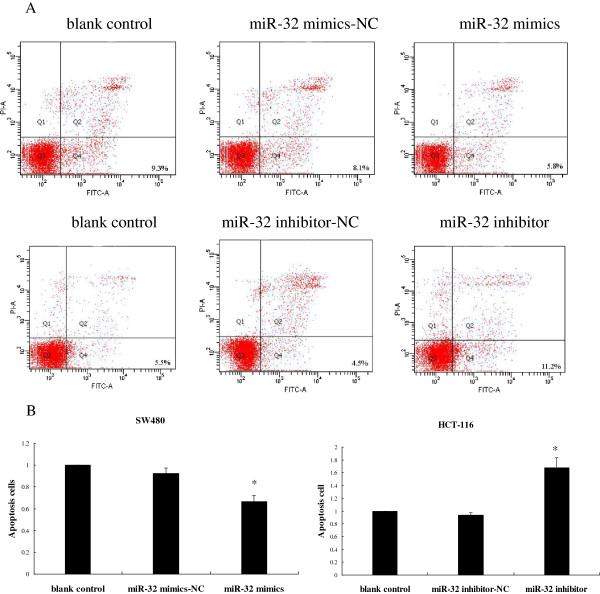
**Apoptosis assay.** (**A**) Percentage of apoptotic cells out of total measured cell population was seen in the bottom right quadrant. A representative experiment of three performed was shown. (**B**) The results were expressed as fold change relative to the corresponding blank control. *P < 0.05, as compared miR-32 mimics or inhibitor with other two corresponding groups. Data were the means of three measurements and the bars represented SD of the mean.

To measure the effect of miR-32 on CRC cell apoptosis, 72 h after transfection, apoptosis was measured at 72 h after miR-32 transfection or miR-32 inhibitor treatment, by flow cytometry. Annexin V-FITC(+) apoptotic cells were significantly decreased in miR-32 mimics transfected group compared to NC or blank control. The percentage of apoptotic cells in the miR-32 inhibitor treated group was higher than he other two control groups (Figure 5). The findings indicated the anti-apoptotic role in CRC cells.

### MiR-32 promoted CRC cell migration and invasion

**Figure 6:**
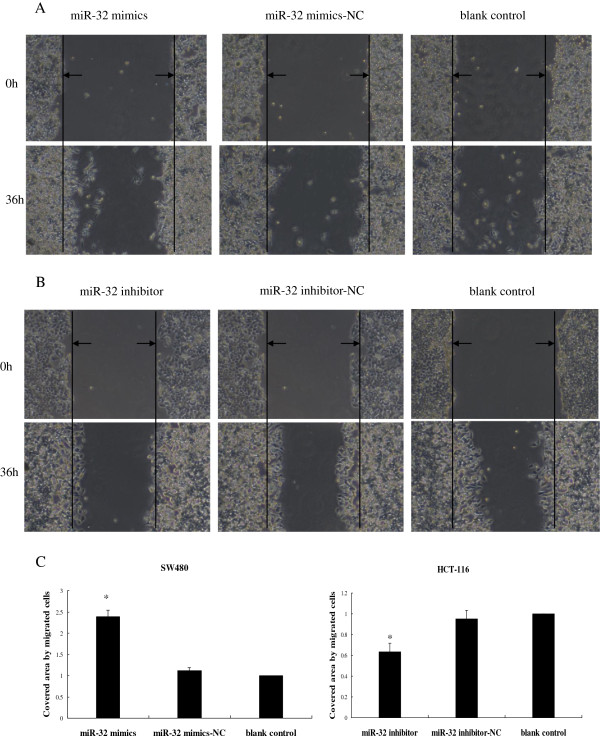
**Wound healing assay.** SW480 and HCT-116 cells were transfected with miR-32 mimics and miR-32 inhibitor, respectively. Movement of cells into wound was shown for miR-32 mimics (**A**), and miR-32 inhibitor-transfected (**B**) cells at 0 and 36 h post scratch (40×). The arrows indicated the boundary lines of scratch. Cell migration was assessed by recover of the scratch. The area of the wound was measured at the two time points in every group, and % reduction of initial scratch area was compared. *P < 0.05, as compared miR-32 mimics or inhibitor with other two corresponding groups. (**C**) The results were expressed as fold change relative to the corresponding blank control. Data were the means of three measurements and the bars represented SD of the mean.

**Figure 7:**
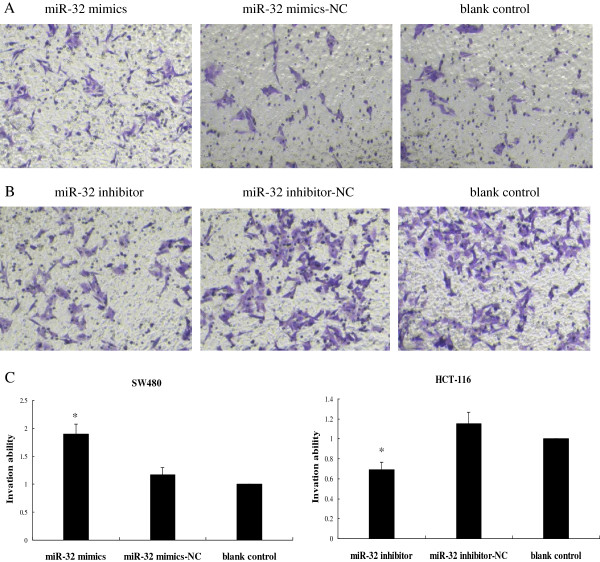
**Transwell invasion assay.** SW480 and HCT-116 cells were transfected with miR-32 mimics and miR-32 inhibitor, respectively. (**A**, **B**) CRC cells penetrating the membrane were fixed and 0.1% crystal violet stained after 24 h as described in experimental procedures. (**C**) CRC cells penetrating the membrane were represented as the fold over blank control. *P < 0.05, as compared miR-32 mimics with its other two groups, or miR-32 inhibitor with other two groups. Data were the means of three measurements and the bars represented SD of the mean.

To evaluate the impact of miR-32 on cell migration and invasion, the wound healing assay and matrigel invasion assay were employed. We found that overexpression of miR-32 induced SW480 cell migration (Figure 6A), whereas its knock-down inhibited HCT-116 cell migration (Figure 6B). Consistent with this finding, matrigel invasion assay showed that miR-32 overexpression significantly enhanced invasion capacity of SW480 cells (Figure 7A), while knock-down of miR-32 inhibited invasion in HCT-116 cells (Figure 7B). These observations suggested that miR-32 played an important role in promoting migration and invasive potential of CRC cells.

## Discussion

Identification of cancer-specific miRNAs and their targets is critical for understanding their roles in tumorigenesis, and may be important for finding out novel therapeutic targets. The expression of miR-32 has been shown to be upregulated in diverse types of malignancies, e.g. kidney cancer and prostate cancer [23,24], and recently miR-32 was shown to be androgen-regulated and overexpressed in castration-resistant prostate cancer. MiR-32 has also been demonstrated to reduce apoptosis by targeting B-cell translocation gene 2 (BTG2), a transcriptional cofactor that has antiproliferative properties [25]. Gocek et al. [26] also reported that miR-32 blockade was sufficient to elevate proapoptotic factor Bim expression and sensitize acute myelogenous leukemia (AML) cells to chemotherapy-induced apoptosis. These data underline a fundamental role of this miRNA as an oncogene. Currently, there are accumulating evidences that the aberrant expression of miRNAs is linked to the development of CRC [15,27]. Using a miRNA microarray analysis, it has been reported that miR-32 is significantly upregulated in CRC [15]. However, the function of miR-32 in CRC carcinogenesis remains unknown.

In this study we investigated the function and possible mechanisms of miR-32 in regulating some biological properties of CRC cells. First, we found that endogenous miR-32 expression is relatively high in low-differentiated HCT-116 cells and low in differentiated HT-29 cells. We also found that its expression is lower in low metastatic ability SW480 cells than in high metastatic ability SW620 cells. This expression pattern raises that possibility that miR-32 is related to some CRC biological properties.

Based on the miR-32 expression level, we chose SW480 and HCT-116 cells for the subsequent gain-of-function and loss-of-function studies, respectively. Our results supported that miR-32 promoted CRC cells growth, migration, and invasion and reduces apoptosis in vitro. On the other hand, downregulation of miR-32 in CRC was related to its inhibition. To address the molecular mechanisms involved in miR-32-mediated biological properties change, PTEN was selected for further study because it was predicted to be a target of miR-32 by bioinformatics analysis. The PTEN gene has been identified as a tumor suppressor gene located on human chromosome region 10q23 [28]. The key target of PTEN is phosphatidylinositol 3, 4, 5-trisphosphate (PIP3) [29], the direct product of phosphatidylinositol 3-kinase (PI3K). The PTEN/PI3K/Akt pathway is highly involved in tumorigenesis. PTEN has been shown to inhibit tumor cell growth and invasion by blocking the PI3K/Akt pathway [30]; it can dephosphatize PI3K at the 3-phosphate site and negatively regulates the Akt signal pathway. Akt regulates cell growth and inhibits apoptosis via controlling downstream proteins [21]. Thus, alteration of PTEN facilitates cell proliferation, invasion, migration, and angiogenesis [31-33] and inhibits apoptosis [34-36]. Loss of nuclear PTEN expression was found to be associated with liver metastasis, and reduced PTEN expression predicts local recurrence in CRC [37]. PTEN expression status also predicts responsiveness to cetuximab therapy, which targets the epidermal growth factor receptor signal pathway [38]. Hence, it is an attractive target for anti-cancer therapy.

Our study showed that PTEN was a possible target of miR-32, and their antagonistic interaction may play a role in the development of CRC. First, the luciferase reporter assay demonstrated its downregulation was mediated by the direct binding of miR-32 to the PTEN 3^′^-UTR, because the alteration of this region abolished this effect. Secondly, overexpression of miR-32 suppressed PTEN protein levels without any change in PTEN mRNA expression, and vice versa. Therefore, we proposed that the main mechanism of miR-32-induced PTEN suppression was post-transcriptional. Finally, overexpression of miR-32 led to increased cell proliferation, migration, invasion and reduced apoptosis in CRC cells.

Our results provided the first insight into the function of miR-32 in regulating some biological properties of CRC cells, at least in part by targeting the anti-oncogene PTEN, highlighting the function of miRNA in the process of tumor progression.

## Conclusions

In conclusion, the present study demonstrated previously uncharacterized biological functions of miR-32 in CRC cells In addition, PTEN was negatively regulated at the posttranscriptional level by miR-32 via a binding site of PTEN-3^′^-UTR. These findings suggested that miR-32 was possibly involved in tumorigenesis of CRC at least in part by suppression of PTEN. And miR-32 was a potential candidate for miRNA-based therapy against CRC.

## Material and methods

### Cell culture and reagents

The CRC cell lines HT-29, HCT-116, LOVO, SW480, and SW620 were cultured in RPMI-1640 medium (Gibco, USA) supplemented with 10% fetal bovine serum (FBS; Gibco, USA), 100 IU/ml penicillin and 100 μg/ml streptomycin in humidified 5% CO_2_ at 37°C. MiR-32 mimics, miR-32 mimics negative control (miR-32 mimics-NC), miR-32 inhibitor, and miR-32 inhibitor negative control (miR-32 inhibitor-NC) were purchased from Ribobio (RiboBio Co. Ltd., China).

### Real-time quantitative RT-PCR (qRT-PCR)

To quantitate miRNA expression, total RNA was extracted from CRC cell lines with RNAiso Plus (Takara, Japan). The isolated total RNA was reverse transcribed using the One Step PrimeScript® miRNA cDNA Synthesis Kit (Takara, Japan) according to the manufacturer’s instructions. Relative expression was calculated via the comparative cycle threshold (Ct) method using the expression of U6 small nuclear RNA as the reference. The sequence-specific forward primers for mature miR-32 and U6 internal control were 5^′^- CGGTATTGCACATTACTAAGTTGCA -3^′^ and 5^′^- CTCGCTTCGGCAGCACA-3^′^, respectively. The Uni-miR qPCR Primer was included in the kit. The amount of miRNA was monitored with SYBR® Premix Ex Taq™ II (Perfect Real Time) (Takara, Japan). The reactions were performed on a LightCycler® (Roche Diagnostics, USA). The PCR conditions were 30s at 95°C, followed by 40 cycles at 95°C for 5 s and 60°C for 20s. The 2^-△Ct^(2^-[(Ct of gene) -(Ct of U6)]^) method was used for analysis.

### Cell transfection

The miR-32 gain-of-function study was performed using miR-32 mimics (100 nM) and its negative control (100 nM) on the SW480 cell line. The loss-of-function study was performed with miR-32 inhibitor (150 nM) and its negative control (150 nM) on the HCT-116 cell line. For each cell line, there was a blank control without any transfection. Cells were transfected using lipofectamine™ 2000 reagent (Invitrogen, USA) in Opti-MEM (Gibco, USA), according to the manufacturer’s instructions. The relative level of miR-32 in transfected cells was examined by qRT-PCR.

### Dual-luciferase reporter assay

The region of human PTEN-3^′^UTR, generated by PCR amplification, was cloned into the pmiR-RB-REPORT™ luciferase reporter plasmid (RiboBio Co. Ltd., China). The primers selected were: PTEN-3^′^UTR-wt-F: 5^′^-CCGCTCGAGTTATTATTTTTCCTTTGGAATGTGAAGG- 3^′^, PTEN-3^′^UTR-wt-R: 5^′^-GAATGCGGCCGCTGACAAGAATGAGACTTTAATCAGTTTT -3^′^, PTEN-3^′^UTR-mut-F: 5^′^- ATTTTGCTCCTAATTGTTCATAACGATGGCTG -3^′^, PTEN-3^′^UTR-mut-R: 5^′^-TGAACAATTAGGAGCAAAATTTCTAGAACTAAACATT-3^′^. These constructs were named pmiR-PTEN-wt and pmiR-PTEN-mut. For the reporter assay, SW480 cells were plated onto 24-well plates and transfected with 500 ng of pmiR-PTEN-wt or pmiR-PTEN-mut and 100 nM miR-32 mimics or NC using lipofectamine 2000. After transfection for 48 h, cells were harvested and assayed with the Dual-Luciferase Reporter Assay System (Promega, USA) according to the manufacturer’s instructions. The tests were repeated in triplicate.

### qRT-PCR for the miR-32 and PTEN mRNA

Transfected cells were incubated 48 h before RNA extraction. qRT-PCR for miR-32 after transfection was performed as previously described. For PTEN, total RNA was reverse transcribed using the PrimeScript® RT Master Mix Perfect Real Time (Takara, Japan). PTEN mRNA level was normalized to housekeeping gene β-actin with the following primers: PTEN forward 5^′^- AAAGGGACGAACTGGTGTAATG -3^′^, and reverse 5^′^- TGGTCCTTACTTCCCCATAGAA -3^′^; β-actin forward 5^′^- GGCGGCAACACCATGTACCCT -3^′^, and reverse 5^′^-AGGGGCCGGACTCGTCATACT-3^′^. Changes in the expression were calculated using the 2^-△Ct^ method.

### Western blot

Transfected cells were harvested for immunoblot analysis after 72 h incubation. Cells were lysed in lysis buffer (Beyotime, China), and protein concentrations were measured using the BCA protein assay kit (Beyotime, China). Total protein was separated by SDS-PAGE using a 12% polyacrylamide gel and electroblotted onto a polyvinylidenefluoride membrane (PVDF; Millipore, USA). The membrane was immunoblotted overnight at 4°C with primary antibodies: rabbit monoclonal antibody against human PTEN (1:500 dilution; Cell Signaling Technology, USA), mouse monoclonal antibody against human β-actin (1:2000 dilution; Beyotime, China). A secondary antibody, horseradish peroxidase-conjugated goat IgG (1:1000 dilution; Beyotime, China), was incubated with the membrane for 1 h after 3 washes with TBST. Signals were detected with ECL detection reagent (Beyotime, China). The images were obtained on Kodak film and quantified by Quantity One (Bio-Rad, USA). All experiments were performed in triplicate.

### MTT assay

Viable cell numbers were measured with 3-(4,5-dimethylthiazol-2-yl)-2, 5-diphenyltetrazolium bromide (MTT) assay. SW480 or HCT-116 cells were plated in 96-well plates and incubated for 24, 48, 72, 96 h respectively after transfection. 20 μl of 5 mg/mL MTT (Sigma,USA) was added into each corresponding test well, and incubated for 4 h in 37°C incubator. The supernatant was then discarded, and 200 μl of DMSO (dimethyl sulfoxide)was added to each well to dissolve the formazan. Optical density (OD) was evaluated by measuring the absorbance. The absorbance at 570 nm (A570) of each well was read on a spectrophotometer. All experiments were performed in triplicate.

### Apoptosis assay

The apoptosis ratio was analyzed using the Annexin V-FITC Apoptosis Detection Kit (Beyotime, China). At 72 h after transfection cells were harvested and resuspended in binding buffer containing Annexin V-FITC and PI according to the manufacturer’s instructions. The samples were analyzed by flow cytometry (FACScan; BD Biosciences, USA). Cells were discriminated into viable cells, necrotic cells, and apoptotic cells by using BD FACSDiva 6.1.3 software (BD Biosciences, USA), and then the percentages of apoptotic cells from each group were compared. Tests were repeated in triplicate.

### Wound healing assay

SW480 cells or HCT-116 cells were seeded onto 6-well plates. When the cell confluence reached about 80% and above at around 48 h post-transfection, scratch wounds were made by scraping the cell layer across each culture plate using the tip of 10 μl pipette. After wounding, the debris was removed by washing the cells with PBS. Wounded cultures were incubated in serum-free medium for 36 h, and then 3 fields (40×) were randomly picked from each scratch wound and visualized by microscopy to assess cell migration ability. The experiments were performed in triplicate.

### In vitro transwell invasion assay

Transwell membranes (polycarbonic membrane, diameter 6.5 mm, pore size 8 μm) (Corning Costar, USA) coated with Matrigel (BD Biosciences, USA) were used to assay cell invasion in vitro. At 48 h post-transfection, cells were resuspended into serum-free medium. Transfected cells (10 × 10^4^ in 200 μl serum-free medium) were reseeded into the upper chamber, and 0.6 ml medium with 10% FBS was added to the lower chamber as chemoattractant. After 24 h incubation, non-invading cells on the upper surface of the membrane were removed with a cotton swab. The invasive cells, which penetrated to the lower surface, were fixed with 4% paraformaldehyde and stained with 0.1% crystal violet (Beyotime, China). The number of cells invading the membrane was counted from 5 randomly selected visual fields with an inverted microscope at 100× magnification. Data were obtained from 3 independent experiments.

### Statistical analysis

Experimental data were presented as the mean ± standard deviation (SD). All statistical analyses were performed using *T*-test when only 2 groups were compared, and by ANOVA when 3 or more groups were compared. All analyses were performed with SPSS 19.0 (SPSS Inc., USA), and a value of *P* < 0.05 was considered to indicate statistical significance.

## Abbreviations

miR: microRNAs, miRNAs; PTEN: phosphatase and tensin homologue; CRC: colorectal carcinoma; 3′-UTR: 3^′^-untranslated region; NC: negative control; FBS: fetal bovine serum.

## Competing interests

The authors declare that they have no competing interests.

## Authors’ contributions

WYW and YZ designed research and analyzed data. WYW, JFY, XF, HW, SCY, PCY, WKT and GLW carried out molecular biology studies. WYW and YZ wrote the paper. All authors have read and approved the final manuscript.
